# *Ex Vivo* Infection of Human Skin Models with Herpes Simplex Virus 1: Accessibility of the Receptor Nectin-1 during Formation or Impairment of Epidermal Barriers Is Restricted by Tight Junctions

**DOI:** 10.1128/jvi.00262-23

**Published:** 2023-06-08

**Authors:** Nydia C. De La Cruz, Maureen Möckel, Hanna Niehues, Matthias Rübsam, Wolfram Malter, Max Zinser, Claude Krummenacher, Dagmar Knebel-Mörsdorf

**Affiliations:** a Center for Biochemistry, University Hospital Cologne, University of Cologne, Cologne, Germany; b Department of Pediatrics, University Hospital Cologne, University of Cologne, Cologne, Germany; c Department of Dermatology, Radboud University Medical Center, Radboud Institute for Molecular Life Sciences, Nijmegen, The Netherlands; d Department Cell Biology of the Skin, University Hospital Cologne, University of Cologne, Cologne, Germany; e Cologne Excellence Cluster on Cellular Stress Response in Aging-associated Diseases, University Hospital Cologne, University of Cologne, Cologne, Germany; f Department of Gynecology and Obstetrics, University Hospital Cologne, University of Cologne, Cologne, Germany; g Department of Plastic, Reconstructive and Aesthetic Surgery, University Hospital Cologne, University of Cologne, Cologne, Germany; h Department of Biological and Biomedical Sciences, Rowan University, Glassboro, New Jersey, USA; University of Arizona

**Keywords:** HSV-1, nectin-1, epidermal equivalents, N/TERT-1 cells, primary human keratinocytes, nonlesional atopic dermatitis skin, human skin, virus entry, epidermal barriers, IL-4/IL-13, human keratinocytes, nonlesional atopic dermatis keratinocytes, tight junctions

## Abstract

Herpes simplex virus 1 (HSV-1) must overcome epidermal barriers to reach its receptors on keratinocytes and initiate infection in human skin. The cell-adhesion molecule nectin-1, which is expressed in human epidermis, acts as an efficient receptor for HSV-1 but is not within reach of the virus upon exposure of human skin under nonpathological conditions. Atopic dermatitis skin, however, can provide an entry portal for HSV-1 emphasizing the role of impaired barrier functions. Here, we explored how epidermal barriers impact HSV-1 invasion in human epidermis and influence the accessibility of nectin-1 for the virus. Using human epidermal equivalents, we observed a correlation of the number of infected cells with tight-junction formation, suggesting that mature tight junctions prior to formation of the stratum corneum prevent viral access to nectin-1. Consequently, impaired epidermal barriers driven by Th2-inflammatory cytokines interleukin 4 (IL-4) and IL-13 as well as the genetic predisposition of nonlesional atopic dermatitis keratinocytes correlated with enhanced infection supporting the impact of functional tight junctions for preventing infection in human epidermis. Comparable to E-cadherin, nectin-1 was distributed throughout the epidermal layers and localized just underneath the tight-junctions. While nectin-1 was evenly distributed on primary human keratinocytes in culture, the receptor was enriched at lateral surfaces of basal and suprabasal cells during differentiation. Nectin-1 showed no major redistribution in the thickened atopic dermatitis and IL-4/IL-13-treated human epidermis in which HSV-1 can invade. However, nectin-1 localization toward tight junction components changed, suggesting that defective tight-junction barriers make nectin-1 accessible for HSV-1 which enables facilitated viral penetration.

**IMPORTANCE** Herpes simplex virus 1 (HSV-1) is a widely distributed human pathogen which productively infects epithelia. The open question is which barriers of the highly protected epithelia must the virus overcome to reach its receptor nectin-1. Here, we used human epidermal equivalents to understand how physical barrier formation and nectin-1 distribution contribute to successful viral invasion. Inflammation-induced barrier defects led to facilitated viral penetration strengthening the role of functional tight-junctions in hindering viral access to nectin-1 that is localized just underneath tight junctions and distributed throughout all layers. We also found nectin-1 ubiquitously localized in the epidermis of atopic dermatitis and IL-4/IL-13-treated human skin implying that impaired tight-junctions in combination with a defective cornified layer allow the accessibility of nectin-1 to HSV-1. Our results support that successful invasion of HSV-1 in human skin relies on defective epidermal barriers, which not only include a dysfunctional cornified layer but also depend on impaired tight junctions.

## INTRODUCTION

Virus entry in cells is mostly well studied with respect to virus-receptor interactions; the challenge, however, is to understand how these findings relate to entry events *in vivo*, and how viruses gain access to their receptors on target cells in tissue. Herpes simplex virus 1 (HSV-1) penetrates the human host organism via mucosal surfaces or skin and establishes productive infection largely in the epithelium, which is followed by latent infection in sensory ganglia for the life of the host. Cellular entry of HSV-1 depends on the interaction between components of the host cellular membranes and viral glycoproteins (1, 2). The cell-adhesion molecule nectin-1 serves as the primary receptor for HSV-1 to infect human epithelial cells and neurons, while herpesvirus entry mediator (HVEM) and modified 3-O-sulfated-heparan sulfate represent further receptors (3–5). HSV-1 binds to nectin-1 via its envelope glycoprotein D (gD), which is essential for fusion with a cellular membrane to occur (6). Infection studies in nectin-1- or HVEM-deficient epidermis identified nectin-1 as the major receptor in murine epidermis, while HVEM has a more limited role (7).

Nectin-1 belongs to the calcium-independent immunoglobulin superfamily of adhesion molecules comprising four members, all of which can transinteract with each other through their extracellular domains and form various cell-cell adhesion complexes (8). Nectins initially form cis-homo dimers, which then undergo lateral cluster formation on the cell surface followed by transinteraction with nectin clusters on the opposing cell surface (9). The cytoplasmic tail of nectins bind afadin, an F-actin-binding protein. Together with cadherin-catenin complexes, the nectin-afadin complexes constitute adherens junctions (AJ), which are characterized by different adhesive properties of cadherins and nectins (10). The formation of nectin-afadin complexes precedes the assembly of cadherin-catenin complexes at AJs, and the presence of AJs, in turn, is a prerequisite for the formation of tight junctions (TJ). While AJs provide the mechanical connection of adjacent cells, TJs present in the most apical viable epidermal layer act as barriers, which control the paracellular transport of molecules. The minimal TJ complex comprises the integral membrane proteins occludin and claudin-1 and the scaffolding protein ZO-1 which links TJs to the actin cytoskeleton. While occludin and ZO-1 localize in the apical granular layer of the epidermis, claudin-1 is also present outside TJs and distributed throughout the suprabasal epidermal layers (11).

Upon exposure of human skin to HSV-1, the virus must overcome multiple barriers to reach its receptor nectin-1 in the epidermis. The initial epidermal barrier is based on antimicrobial peptides and a physical protection provided by the uppermost stratum corneum with its lipid-sealed cell-cell contacts. Together with the stratum corneum, TJs form the physical barriers of the epidermis. *Ex vivo* infection studies of human skin and oral mucosa confirm that HSV-1 cannot penetrate skin or mucosa via the external surface; only when the dermis is separated, the virus can gain access to the epidermis or oral epithelium via the basal layer and infect both undifferentiated and differentiated keratinocytes (12, 13). Conditions that allow the virus to overcome the epidermal barriers of human skin could be mechanical injuries resulting in epithelial breaks or pathological skin conditions leading to impaired barrier functions. Unexpectedly, the wounded human skin surface does not allow penetration of HSV-1 upon *ex vivo* infection (13). However, we observed successful invasion in lesional atopic dermatitis skin, which is characterized by inflammation-induced modifications and dysfunctional physical barriers (14). Patients with atopic dermatitis can be seriously affected by HSV skin infections termed eczema herpeticum (15, 16). We also demonstrated that induced Th2 immune responses, which mimic an atopic dermatitis-like phenotype, can lead to successful viral penetration as shown by infected cells in interleukin-4 (IL-4)/IL-13-treated human skin (14). Thus, we conclude that HSV-1 opportunistically relies on skin conditions that facilitate the accessibility of nectin-1. The open questions are whether and how the impairment of intercellular junctions influences the presence and localization of nectin-1 so that the receptor can be reached by HSV-1.

Here, we explored how formation of the TJ barrier contributes to viral accessibility of nectin-1 in the human epidermis by using human epidermal equivalents derived from N/TERT-1 cells or primary human keratinocytes. As human epidermal equivalents permit terminal differentiation of keratinocytes which then resemble features of fully stratified epidermis (17), this model allows the analysis of HSV-1 infection and nectin-1 distribution during differentiation and functional barrier formation. Differentiation includes extensive reorganization of intercellular junctions, thus the accessibility of nectin-1 to HSV-1 might differ during junction formation. Our focus was on how the viral receptor nectin-1 is distributed on keratinocytes during the differentiation process and under pathological skin conditions. Furthermore, we investigated how inflammation-induced impairment of epidermal barriers can influence the accessibility of nectin-1 for HSV-1 in human skin and whether the facilitated viral invasion in atopic dermatitis skin relies on the redistribution of the receptor.

## RESULTS

### Susceptibility of N/TERT-1 cells to HSV-1 during epidermal barrier formation.

To explore how barrier formation impacts HSV-1 invasion during differentiation, we utilized human epidermal equivalents derived from the keratinocyte cell line N/TERT-1. Human N/TERT-1 keratinocytes were shown to retain differentiation and barrier characteristics of primary keratinocytes in organotypic skin models and can be used to generate an epidermal equivalent with atopic dermatitis features (18, 19). We confirmed the efficient infection of N/TERT-1 cells by HSV-1 in culture as shown recently (20) and demonstrated expression of the receptor nectin-1 on nearly all cells by flow cytometry (data not shown).

**FIG 1:**
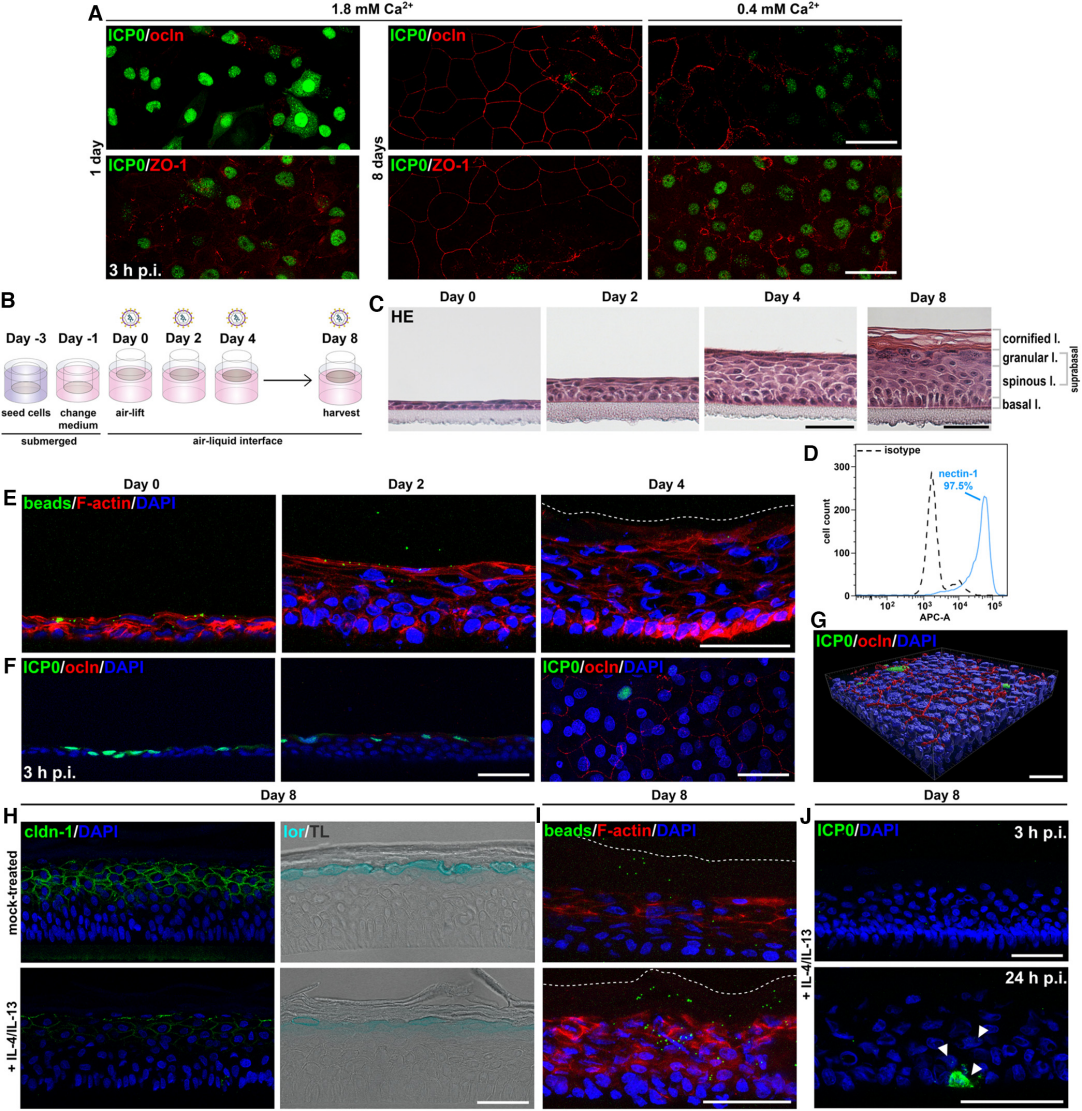
HSV-1 penetration in human epidermal equivalents based on N/TERT-1 cells. (A) N/TERT-1 cells under differentiating (1.8 mM Ca^2+^) and nondifferentiating (0.4 mM Ca^2+^) conditions were infected with HSV-1 at a multiplicity of infection (MOI) of 20 PFU/cell. ICP0-expressing cells (green) correlated with the absence or discontinuous occludin (ocln; red) and diffuse ZO-1 (red) stainings after 1 day of differentiation and 8 days under low calcium (0.4 mM Ca^2+^). In contrast, cultures differentiated for 8 days with distinct occludin (red) or ZO-1 stainings (red) at suprabasal cells only show single infected cells (*n* > 3 independent experimental settings). (B) Schematic illustrating the generation of 3D cultures on collagen-coated transwell filters and time points of infection. Two days after seeding (day -1), the medium of the submerged cultures was exchanged with 3D differentiation medium. (C) H&E stains depict the development of the 3D cultures with a thin layer of still nucleated corneocytes at day 4 and a fully cornified layer at day 8. (D) Flow cytometry shows nectin-1-positive cells from basal and suprabasal layers of the 3D culture at day 8 (*n* = 2). (E) After incubation with 500-nm beads for 3 h, cross sections indicate uptake of beads (green) in apical cell layers at day 0 and day 2 and only single beads at day 4 (*n* = 3). The dashed line indicates the border of the thin cornified layer. F-actin (red) depicts cell morphology. (F) After infection with HSV-1 at 20 PFU/cell for 3 h, cross sections visualize ICP0-expressing cells (green) in the absence of occludin (ocln; red) prior to airlift (day 0), and few infected cells at day 2 with some punctate occludin staining. Staining of whole mount prepared from a 3D culture at day 4 shows the distribution of cells with punctate occludin (red) staining and single ICP0-expressing cells (green) with a view on the apical surface of the culture (*n* = 3). (G) 3D surface-rendered image of the whole mount shown in panel F visualizes the few ICP0-expressing cells (green) in correlation with punctate occludin (red) at intercellular junctions of the granular layer (*n* = 3). (h) Immunostainings at day 8 show strong decrease of claudin-1 (cldn-1; green) and decreased loricrin (lor; blue) in the granular layer after 7 days of IL-4/IL-13 treatment compared to mock-treated cultures (*n* = 3). Transmission light (TL) visualizes the epidermal layers. (I) After incubation with beads for 24 h, cross sections indicate some beads (green) in the cornified layer of mock-treated cultures and enhanced uptake of beads (green) in the cornified and granular layers after IL-4/IL-13 treatment (*n* = 2). The dashed line indicates the border of the cornified layer. (J) After infection of IL-4/IL-13-treated cultures with HSV-1 at 20 PFU/cell (*n* = 3), no ICP0-expressing cells (green) were observed at 3 hpi and only one small cluster of infected cells (arrowheads) was detected at 24 hpi. DAPI (blue) serves as a nuclear counterstain. Scale bars, 50 μm.

After infection of stratified cultures of various murine knockout keratinocytes, we found that successful HSV-1 entry depends on the extent of impaired TJ integrity (21). Here, we investigated how TJ formation during differentiation of human N/TERT-1 cells influences viral susceptibility by initially employing the *in vitro* differentiation model induced by elevating extracellular calcium levels (22). Under differentiating conditions (1.8 mM Ca^2+^) for 1 day, we observed discontinuous stainings of the TJ components occludin and ZO-1 at the plasma membrane, which were comparable to nondifferentiating conditions (Fig. 1A). In contrast, continuous staining of occludin and ZO-1 was visible after 8 days under differentiating conditions indicating the formation of mature TJs in the apical layer (Fig. 1A). Infected cells were determined by visualizing the immediate early expressed viral protein ICP0 which first localizes in the nucleus and then relocalizes to the cytoplasm during later infection, indicating viral replication (23, 24). Upon infection, ICP0-expressing cells were detected at 3 h postinfection (hpi) under short-term (1 day) differentiation conditions, while only single apical cells with no continuous ZO-1 or occludin stainings were infected after 8 days of differentiation (Fig. 1A). These results support that TJ formation in N/TERT-1 cells can strongly interfere with HSV-1 susceptibility.

Next, we generated human epidermal equivalents with N/TERT-1 cells to explore HSV-1 susceptibility during formation of multiple epidermal barriers in detail (Fig. 1B). Histological sections demonstrated a 1- to 2-layer culture at day 0 with further suprabasal cells after raising the cultures to the air-liquid interface (day 2), indicating early differentiation (Fig. 1C). At day 4, several layers of differentiating cells emerged with a very thin layer of cornification, and at day 8, the cultures were fully differentiated with multiple layers of the stratum corneum (Fig. 1C). To analyze nectin-1 expression on cells from fully stratified epidermal equivalents, we performed flow cytometric analyses, which indicated 97.5% of nectin-1-positive cells representing basal and suprabasal cells (Fig. 1D). The high number of nectin-1-expressing undifferentiated and differentiated N/TERT-1 cells does not mimic the heterogeneous nectin-1 level in human epidermis (13).

The extent of functional barrier formation during the differentiation processes of epidermal equivalents was investigated by penetration assays with latex beads. Penetration of beads was easily detected prior to airlift (day 0) and was still observed at day 2 (Fig. 1E). At day 4, however, only single beads passed the thin layer of cornification, indicating some barrier function of the early cornified layer (Fig. 1E).

Upon infection by HSV-1 prior to airlift (day 0), ICP0 was expressed in many cells at 3 hpi (Fig. 1F). Infection at day 2 resulted in less ICP0-expressing cells compared to day 0 (Fig. 1F), but nearly all apical cells were infected at 9 hpi (data not shown). The delayed efficiency of infection at day 2 correlated with occludin stainings at the membranes of some apical cells, suggesting an early stage of TJ formation, which was not yet apparent at day 0 (Fig. 1F). Infection at day 4 led to single infected cells, which correlated with a strong enrichment of occludin at lateral membranes in the most apical granular layer, as shown by whole-mount stainings (Fig. 1F and G). The 3D image visualizes single apical infected cells in areas with discontinuous occludin staining (Fig. 1G). Our results indicated that TJ formation prior to barrier formation of the stratum corneum strongly interfered with viral invasion. As the initial cornification at day 4 hindered penetration of latex beads, we assume that the thin cornified layer additionally influences the extent of virus invasion.

### Th2-inflammation-induced modifications of N/TERT-1 cultures do not enhance HSV-1 susceptibility.

To further explore the role of TJs and the stratum corneum barrier, we chose conditions that lead to impaired barrier functions. The Th2 cytokines IL-4 and IL-13 were employed in various human skin models to mimic an atopic dermatitis-like Th2-driven inflammation, which results in hyperproliferation, impaired lipid composition of the cornified layer, and reduced skin protein expression; these alterations, in turn, impair keratinocyte barrier functions (25–27). As IL-4/IL-13 treatment of epidermal equivalents based on N/TERT-1 cells can induce histopathological and molecular hallmarks of atopic dermatitis (19), we investigated whether the cytokine-induced barrier alterations enhance HSV-1 invasion. After IL-4/IL-13 addition to the cultures at day 1, we analyzed barrier components after 7 days of treatment (day 8) and observed reduced staining of the terminal differentiation marker loricrin and a strong decrease of the TJ component claudin-1 (Fig. 1H) as described (28) indicating impaired TJs and a dysfunctional epidermal barrier. Effects on differentiation processes were further supported by an aberrant enrichment of F-actin below the granular layer of the cytokine-stimulated cultures (Fig. 1I). Interestingly, penetration assays demonstrated some areas where latex beads invaded the cornified as well as the granular layer after IL-4/IL-13 treatment, which was unlike mock-treated cultures (Fig. 1I). In contrast, infected cells were detected neither in cytokine- (Fig. 1J) nor mock-treated cultures (data not shown) at 3 hpi; only at 24 hpi, one cluster of single ICP0-expressing cells was found after cytokine treatment (Fig. 1J), which was not observed in mock-treated cultures (data not shown). These results suggest that IL-4/IL-13 stimulation alters differentiation processes influencing TJ formation and induces some changes of functional barriers in the cornified layer, however, these changes were insufficient to substantially allow HSV-1 invasion.

### Susceptibility of primary human keratinocytes to HSV-1 in epidermal equivalents.

**FIG 2:**
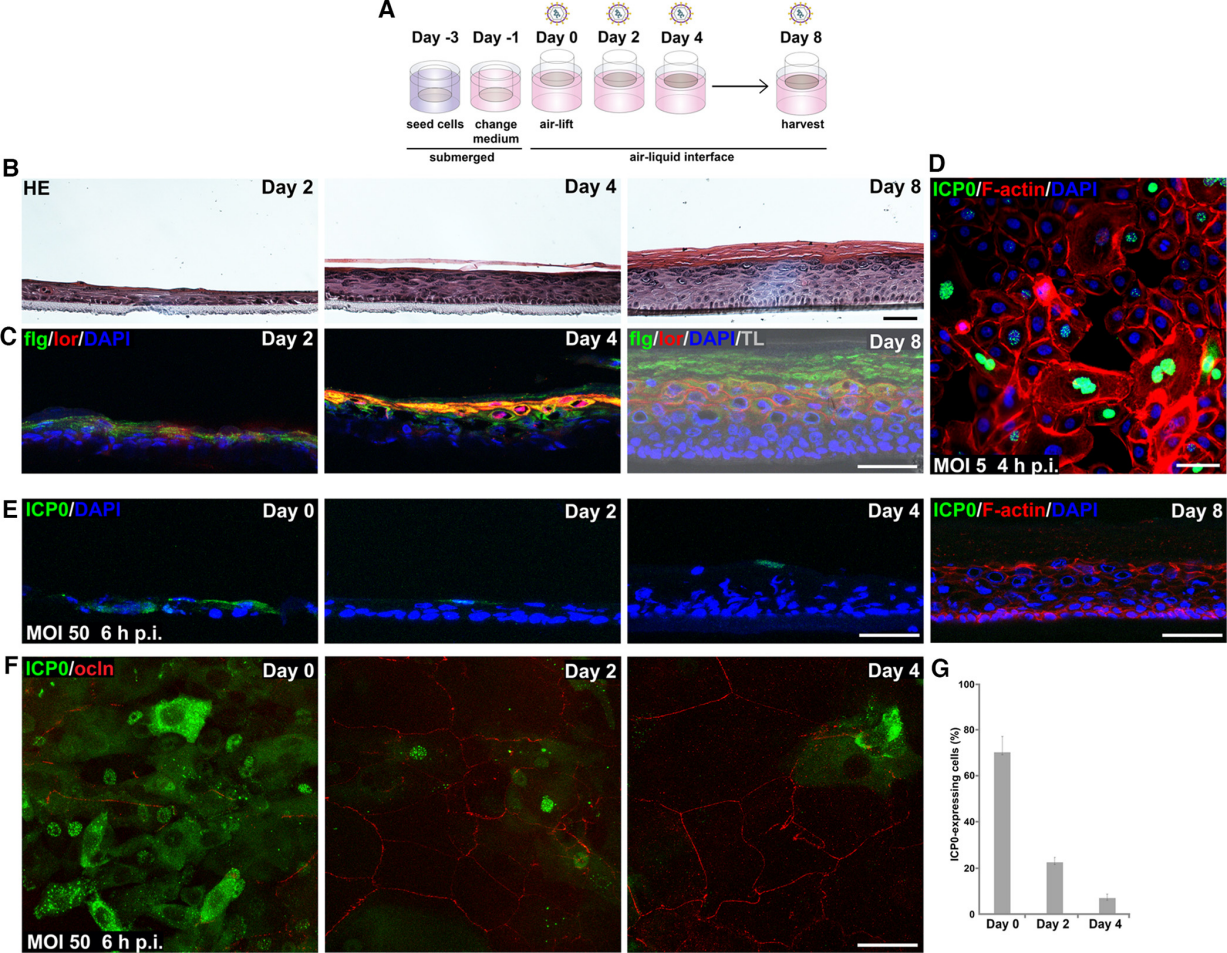
HSV-1 entry in epidermal equivalents based on primary human keratinocytes. (A) Schematic illustrating the generation of 3D cultures on collagen-coated transwell filters and time points of infection. (B) HE-stained sections show the morphology of the developing 3D cultures. (C) Filaggrin (flg; green) and loricrin (lor; red) are detected in the most apical layer at day 2 and 4 and are distributed throughout the cornified and granular layers, respectively, at day 8 (*n* = 2). Transmission light (TL) visualizes the fully cornified layer. (D) As a control, immunostainings demonstrate ICP0-expressing cells (green) after infection of primary human keratinocytes with HSV-1 at 5 PFU/cell for 4 h with F-actin as a cellular counterstain (*n* > 3). (E) After infection with HSV-1 at 50 PFU/cell for 6 h, most cells expressed ICP0 at day 0, small clusters of infected cells were present at day 2, some cells at day 4, and no infected cells at day 8 (*n* ≥ 3). F-actin served as a cellular counterstain. (F) Stainings of whole-mount preparations with view on the apical surface of the cultures visualize the decreasing number of ICP0-expressing cells (green) at day 2 and 4 in correlation with increasing occludin (ocln; red) network in the suprabasal layers. (G) Quantification shows the decreased number of ICP0-expressing cells at day 2 and 4 (*n* = 3). DAPI (blue) serves as a nuclear counterstain. Scale bars, 50 μm.

To dissect barrier formation and successful viral invasion in more detail, we extended our infection studies to epidermal equivalents based on primary human keratinocytes (Fig. 2A) which more closely resemble epidermal homeostasis *in vivo*. The focus was on infection prior to the formation of a fully stratified human epidermis to explore the impact of developing TJs preceding the establishment of a cornified layer. Histological sections demonstrated faster stratification and more heterogeneity compared to N/TERT-1 cells (Fig. 2C). Already at day 2 after airlift, stainings of filaggrin and loricrin, representing barrier components of the squamous layer revealed both markers (Fig. 2D). The histological section at day 4 depicted the initiating cornification, which was supported by the enhanced presence of filaggrin and loricrin in the apical layer (Fig. 2C and D). At day 8, filaggrin and loricrin stainings indicated an intact cornified barrier formation with filaggrin in the stratum corneum and loricrin in the terminally differentiated keratinocytes (Fig. 2D).

Primary human keratinocytes in culture are well infected by HSV-1 (29). When we infected densely grown primary human keratinocytes prior to induced differentiation, we observed approximately 40% of infected cells at 4 hpi (data not shown), visualized in Fig. 2B, which indicated a delayed onset of infection compared to less densely seeded cells and a major delay compared to N/TERT-1 cells. Upon infection of epidermal equivalents prior to airlift (day 0), large areas with most apical but also basal cells infected were observed, while only small clusters of apical cells expressing ICP0 were present at day 2, single cells at day 4, and no infected cells at day 8 (Fig. 2E–G). The strongly decreased number of infected cells at day 2 correlated with areas showing an occludin network of large suprabasal cells (Fig. 2F and G). Thus, we conclude that areas with TJs at day 2 already hamper successful viral invasion while areas with less TJ formation still allow infection. More continuous occludin stainings were observed at day 4 indicating mature TJs, which correlated with infection of only some cells (Fig. 2F). Initiating cornification may additionally contribute to hindered viral accessibility as in N/TERT-1 3D cultures.

**FIG 3:**
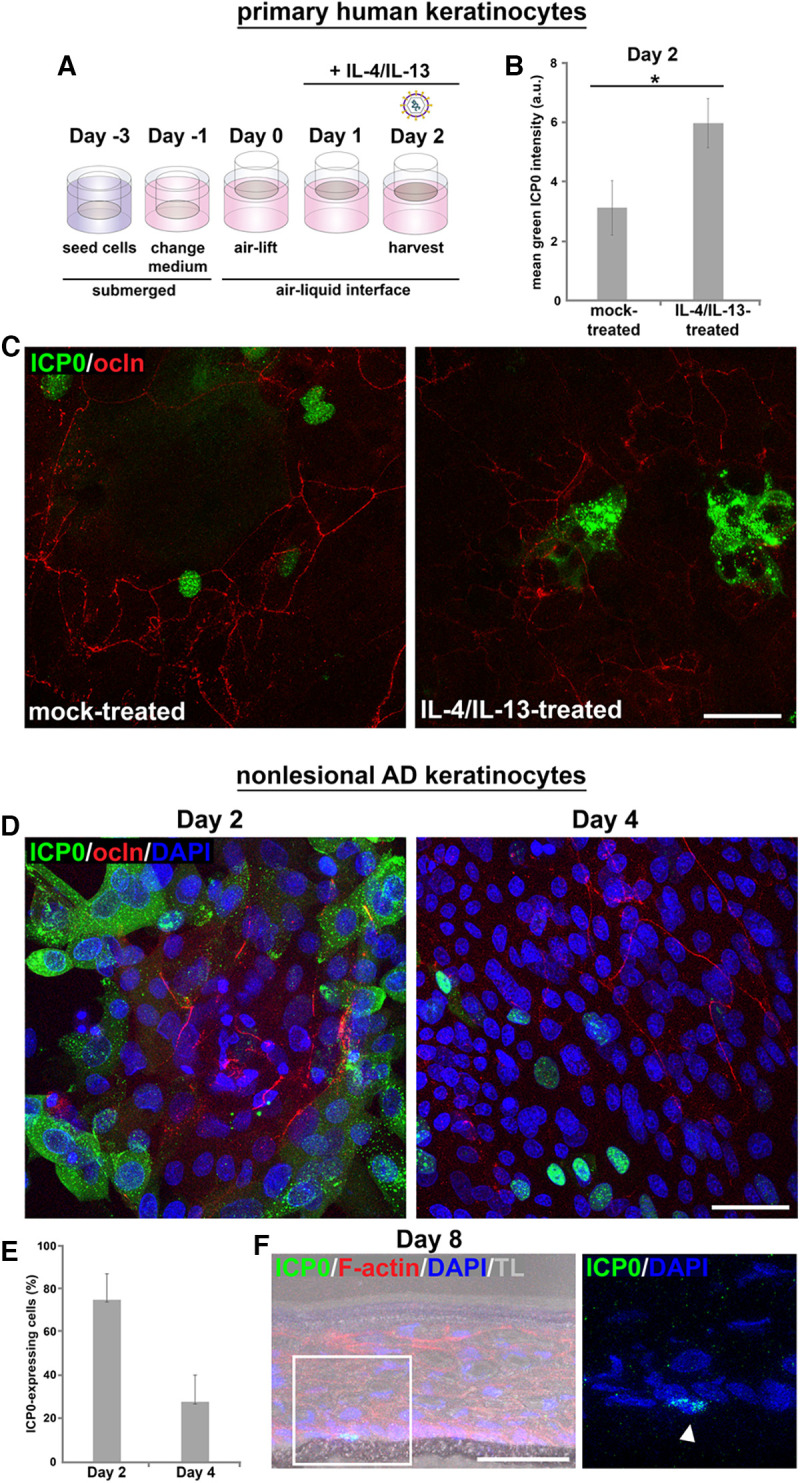
HSV-1 penetration in epidermal equivalents based on primary human keratinocytes treated with IL-4/IL-13 or based on nonlesional atopic dermatitis (AD) keratinocytes. (A) Schematic illustrating the generation of 3D cultures with IL-4/IL-13 treatment at day 1 followed by infection 24 h later. (B) Quantification of fluorescence shows increased ICP0 intensities in IL-4/IL-13-treated cultures at day 2 compared to mock-treated cultures (*n* = 3). *, *P* ≤ 0.05. (C) Max projections of whole mounts prepared at day 2, which were infected with HSV-1 at 50 PFU/cell for 6 h, depict increased staining intensities of ICP0 (green) in IL-4/IL-13- compared to mock-treated cultures (*n* = 3). (D) Whole mounts prepared of 3D cultures based on nonlesional AD keratinocytes were infected with HSV-1 at 50 PFU/cell for 6 h. At day 2, most cells expressed ICP0 (green) except areas with initial occludin (red) network. At day 4, areas with continuous occludin staining enlarged and a reduced number of ICP0-expressing cells was observed (*n* = 3). (E) Quantification shows the decreased number of ICP0-expressing cells at day 4 (*n* = 3). (F) At day 8, single infected cells (green; arrowhead) were visualized (*n* = 3). Transmission light (TL) visualizes the thin cornified layer. F-actin (red) served as a cellular and DAPI (blue) as a nuclear counterstain. Scale bars, 50 μm.

By treating epidermal equivalents with IL-4/IL-13 (Fig. 3A), we further analyzed the effects of early TJ formation on viral susceptibility and addressed whether inflammation-induced changes sufficiently alter TJs prior to the establishment of the cornified layer to enhance HSV-1 invasion. After treatment with IL-4/IL-13 at day 1 followed by infection 24 h later, we observed increased intensities of ICP0 stainings compared to untreated cultures supporting enhanced efficiency of infection (Fig. 3B and C). After the short-term cytokine incubation of 24 h, changes of the occludin network, which was still heterogeneous at this time point (day 2), were difficult to judge. Still, we speculate that in the absence of the stratum corneum, cytokine-induced changes affecting TJ formation correlate with enhanced infection.

To further investigate the impact of defective barriers for HSV-1 invasion, we used primary human keratinocytes of nonlesional atopic dermititis skin for the generation of epidermal equivalents to explore whether genetically induced weakening of epidermal barriers can influence viral invasion. Nonlesional atopic dermatitis skin is characterized by differentiation defects and abnormalities of the stratum corneum (30, 31), although we still know little about genetic barrier defects. Our experiments with epidermal equivalents derived from nonlesional keratinocytes indicated a disordered morphology with a strong delay of TJ and cornified layer formation compared to cultures based on keratinocytes of healthy individuals, supporting an intrinsic predisposition of nonlesional keratinocytes to defective barrier formation. At day 2, we observed most cells infected except small areas with an initial occludin network, which expanded at day 4 when clusters of infected cells were still present (Fig. 3D and E) while only single infected cells were detected at day 8 (Fig. 3F). At this time, the suprabasal layers showed some morphological abnormalities with only initial cornification (Fig. 3F), which was absent at day 4 (data not shown). These results indicated a correlation of delayed and impaired barrier formation, and increased viral invasion, which strengthens our assumption that early TJ formation already interferes with viral infection.

### Distribution of nectin-1 in human epidermal equivalents.

**FIG 4:**
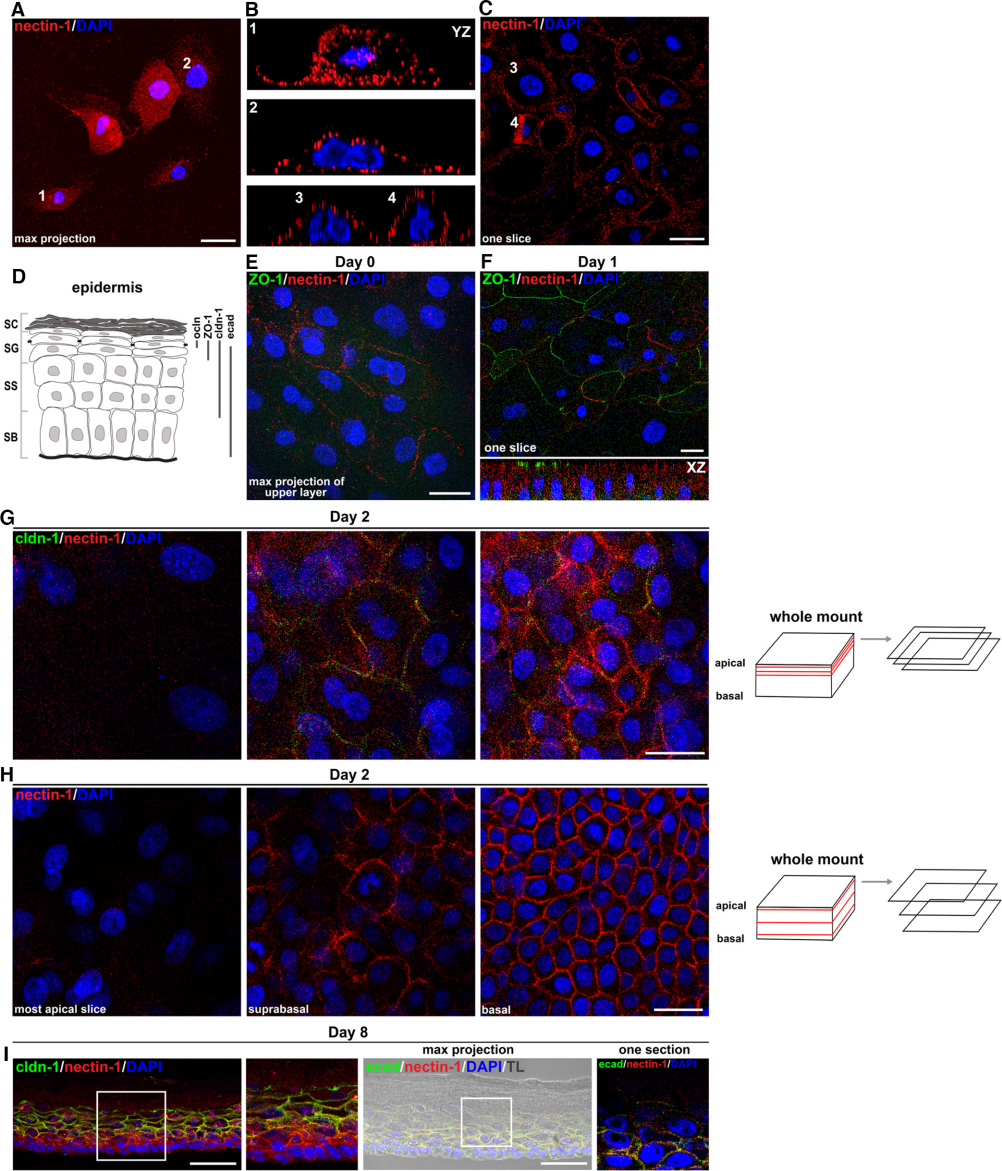
Nectin-1 distribution in primary human keratinocytes and epidermal equivalents. (A) Primary human keratinocytes seeded at low density show nectin-1 (red) localization. (B) YZ images demonstrate equally distributed nectin-1 on basolateral and apical surfaces of cells 1 to 4. (C) Confocal slice of densely seeded keratinocytes visualizes nectin-1 (red) at lateral membranes (D) Schematic illustrating the distribution of the TJ components occludin (ocln), ZO-1, and claudin-1 (cldn-1), and the AJ component E-cadherin (ecad) in the stratum corneum (SC), stratum granulosum (SG), stratum spinosum (SS), and stratum basale (SB). (E) Whole mount of epidermal equivalents prior to airlift (day 0) visualize areas with nectin-1 (red) enriched at lateral membranes in the absence of ZO-1. (F) Apical slice of whole-mount preparation at day 1 show lateral nectin-1 (red) and ZO-1 (green) at the most apical cells. Nectin-1 underneath ZO-1 is shown in the XZ axis. (G) Three apical slices by the apical surface of whole-mount preparations at day 2 demonstrate the absence of nectin-1 (red) and claudin-1 (cldn; green) at the most apical nucleated cells, colocalization of nectin-1 and claudin-1 in the following slices and increased lateral nectin-1 in the third slice. (H) Three slices throughout the whole-mount preparation show the heterogeneous distribution of lateral nectin-1 (red) at suprabasal cells and the strong continuous nectin-1 staining in the basal layer. (I) Cross sections of epidermal equivalents at day 8 show strong nectin-1 (red) in the basal layer and colocalization with claudin-1 (green) in the spinous and granular layer except the most apical granular layer where only claudin-1 is present as shown in the magnification. Colocalization of E-cadherin (ecad; green) and nectin-1 (red) is shown in all epidermal layers. Transmission light (TL) visualizes the morphology of the nucleated and cornified layers. DAPI (blue) serves as a nuclear counterstain. Scale bars, 25 μm (A–H), 50 μm (I).

To reveal how TJs impact the accessibility of nectin-1 to HSV-1, we investigated the localization and distribution of the receptor regarding barrier-forming TJs. When we initially visualized the receptor on primary human keratinocytes in culture, we found nectin-1 all over the cell surface even in densely grown cultures (Fig. 4A–C). This finding correlated with efficient infection of undifferentiated keratinocytes (Fig. 2B). We next investigated how nectin-1 is distributed throughout the layers of epidermal equivalents derived from primary human keratinocytes during differentiation and barrier formation. Prior to airlift (day 0) when most cells were infected (Fig. 2E and F), nectin-1 was present at basal and apical cells with some apical cells showing areas with enriched nectin-1 at lateral membranes; at this early time of differentiation, ZO-1 was rarely visible (Fig. 4E). Intriguingly, at day 1, we observed areas with ZO-1 in addition to nectin-1 enriched at lateral membranes with nectin-1 localizing underneath ZO-1 (Fig. 4F) which supports initial formation of TJs.

We next stained claudin-1, which is distributed throughout the suprabasal layers in fully stratified epidermis (Fig. 4D) to further characterize the localization of TJ components with regard to nectin-1. After taking various slices of the apical layer at day 2, the most apical nucleated cell layer showed neither nectin-1 nor claudin-1, while the cell layers just underneath indicated colocalization of nectin-1 and claudin-1 (Fig. 4G). Enriched nectin-1 at lateral membranes increased from apical to basal layers with strong lateral nectin-1 at all basal cells (Fig. 4G and H). Taken together with the initial occludin network observed at day 2 (Fig. 2F), which correlated with strongly decreased numbers of infected cells (Fig. 2G), we conclude that initial TJ formation can hinder the accessibility of nectin-1, which is present in the various epidermal layers. When we analyzed nectin-1 distribution in fully stratified epidermis (day 8) where no infected cells were found (Fig. 2B), nectin-1 and claudin-1 were present throughout the granular layer (Fig. 4I). Only claudin-1, however, was detected at the most apical nucleated cells (Fig. 4I). Costaining of nectin-1 and E-cadherin confirmed that both adherens junction (AJ) components colocalized in all epidermal layers (Fig. 4I); some areas showed nectin-1 localizing underneath E-cadherin in the most apical granular layer (Fig. 4I, magnification). These results demonstrate that nectin-1 is present at AJs in the granular layer but functional TJs prevent the accessibility of nectin-1 to HSV-1 in human epidermal equivalents. Taken together, nectin-1 is present on all keratinocytes during the differentiation processes while TJs are progressively formed until the cultures are fully stratified which, in turn, correlates with decreasing accessibility of nectin-1 to the virus.

**FIG 5:**
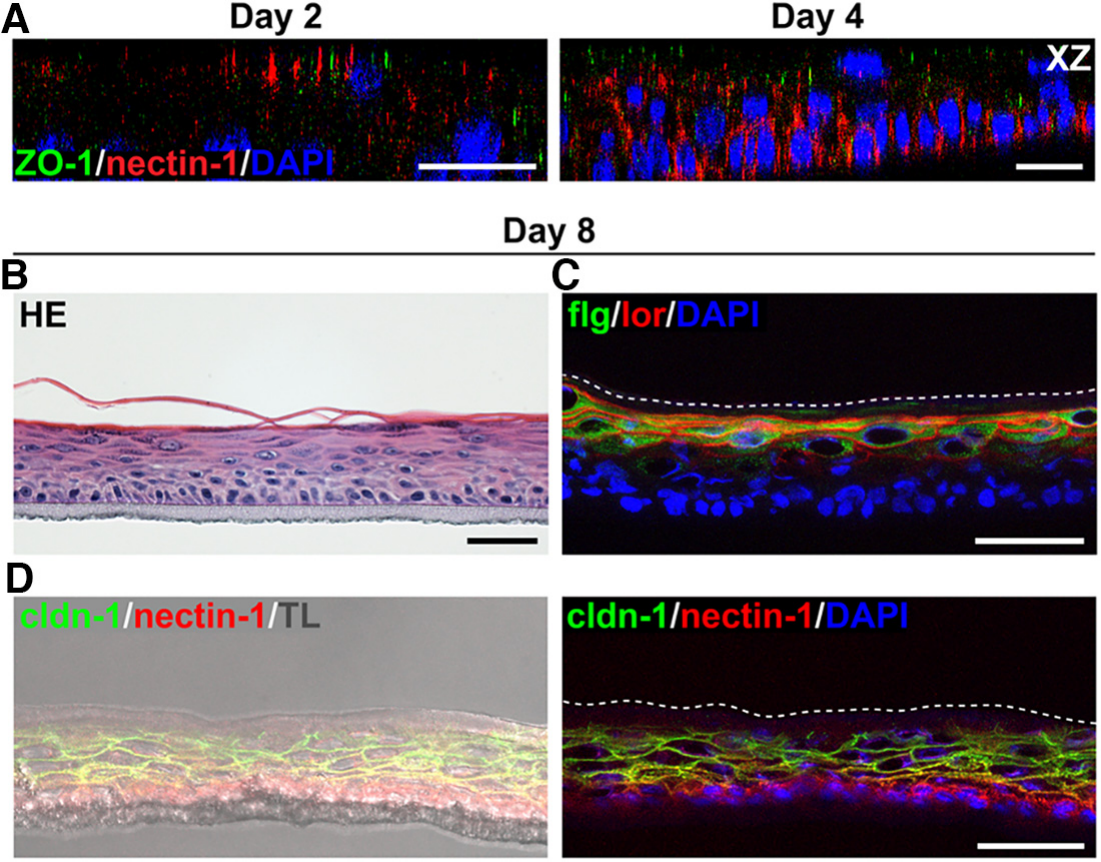
Distribution of nectin-1 in epidermal equivalents based on nonlesional AD keratinocytes. (A) Nectin-1 next to ZO-1 in the apical layer is depicted in the XZ axis. An area with ZO-1 above nectin-1 is shown at day 4. (B) HE-stained section shows the morphology with a thin cornified layer. (C) Filaggrin (flg; green) and loricrin (lor; red) are detected in the most apical nucleated layer. (D) Colocalization of claudin-1 (cldn-1; green) and nectin-1 was present in the suprabasal layers and increased nectin-1 stainings were observed in the basal layer. Transmission light (TL) visualizes the morphology and the dashed line indicates the border of the thin cornified layer. DAPI (blue) serves as a nuclear counterstain. Scale bars, 25 μm (A), 50 μm (B–D).

To investigate whether the defective barriers in epidermal equivalents of nonlesional keratinocytes influence the localization of nectin-1 toward TJs, we visualized the receptor during barrier formation. At day 2, nectin-1 was present next to ZO-1 in the apical layer and only some areas with ZO-1 above nectin-1 were observed at day 4 (Fig. 5A) which correlated with fewer infected cells compared to day 2 (Fig. 3E). These results support that the delayed TJ formation results in better exposure of nectin-1 to the virus compared to 3D cultures of keratinocytes from healthy individuals. Even at day 8, when single infected cells were detected (Fig. 3F), the cultures of nonlesional keratinocytes did not resemble fully stratified epidermis (Fig. 5B) but only showed early cornification, as depicted by the filaggrin and loricrin stainings (Fig. 5C). Claudin-1 and nectin-1 were observed throughout the suprabasal layers while nectin-1 was also strongly present in the basal layer (Fig. 5D). Thus, we conclude that the defective cornified layer in addition to the underdeveloped TJs allows HSV-1 to access single cells.

### Nectin-1 distribution in atopic dermatitis skin and in IL-4/IL-13-treated human skin.

**FIG 6:**
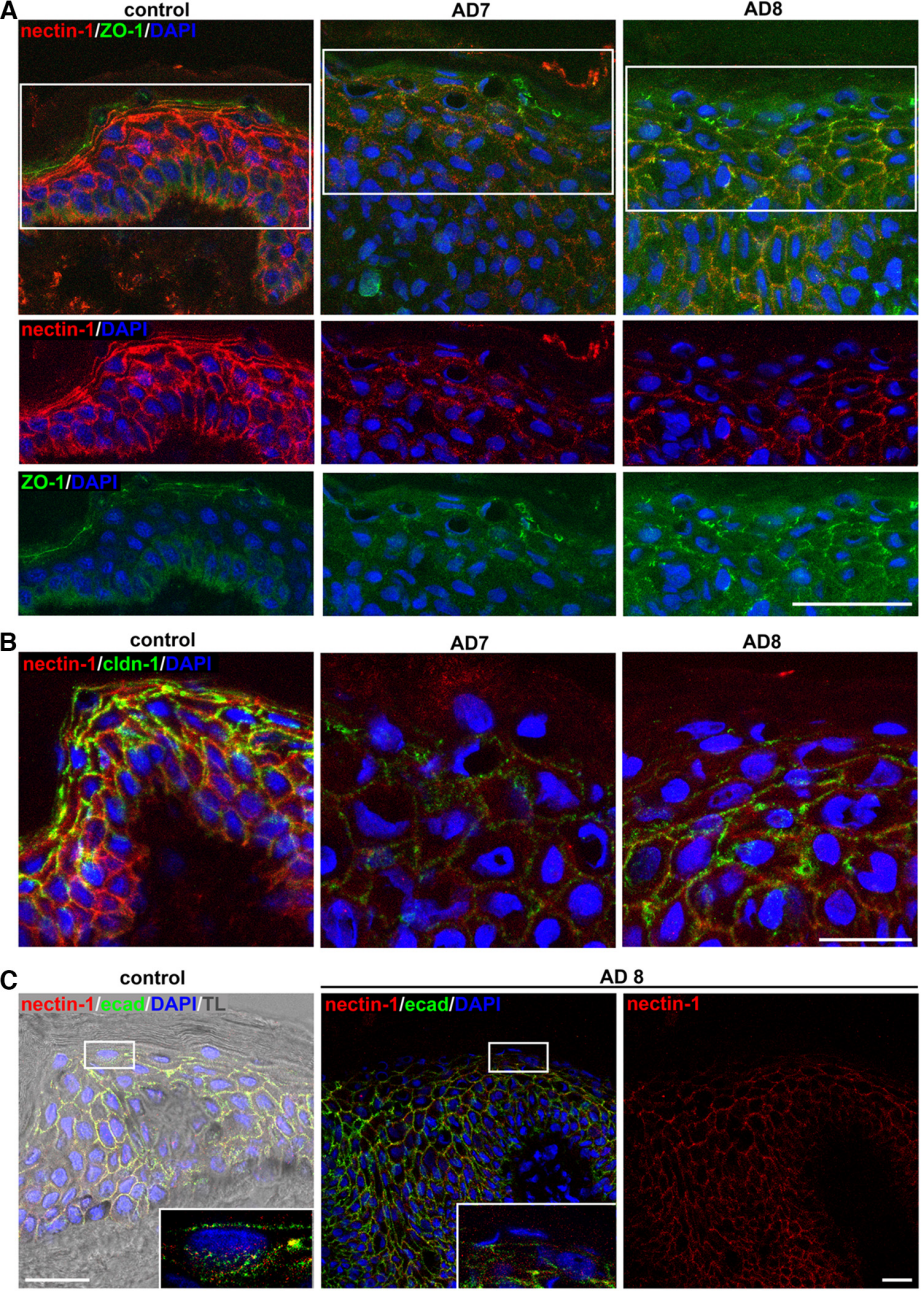
Localization of nectin-1 in atopic dermatitis skin. (A) Cross section of human control skin show nectin-1 (red) throughout the epidermal layers underneath the distinct staining of ZO-1 (green). Cross sections of thickened atopic dermatitis skin (samples AD7 and AD8) demonstrate the redistribution of ZO-1 in the granular and spinous layers compared to control skin. Nectin-1 stainings were diffused in AD7 and AD8 skin samples. (B) Control skin shows distinct claudin-1 in the granular layer while claudin-1 was reduced in AD7 and AD8 with a more punctate staining pattern. Costainings visualize nectin-1 throughout the suprabasal layers both in control and AD7/AD8 skin samples. (C) Colocalization of nectin-1 (red) and E-cadherin (ecad; green) is visible throughout the epidermis in control skin. Colocalization in the apical granular layer is magnified. Transmission light (TL) visualizes the morphology of the epidermis. The staining pattern of E-cadherin and nectin-1 is comparable in control and AD8 skin. DAPI (blue) serves as a nuclear counterstain. Scale bars, 25 μm (B, C), 50 μm (A).

Recent infection studies revealed successful HSV-1 invasion in skin under pathological conditions such as atopic dermatitis skin, which is characterized by various disturbed barrier functions; we demonstrated the redistribution of the TJ markers claudin-1, ZO-1, and occludin in lesional skin samples supporting impaired TJ barriers (14). Here, we addressed whether redistribution of the TJ markers influences the presence and distribution of nectin-1, leading to viral entry in lesional atopic dermatitis skin. In control skin, nectin-1 was present in all epidermal layers, while distinct ZO-1 in the most apical nucleated layer was strictly above nectin-1, as expected (Fig. 6A). In contrast, the discontinuous ZO-1 in the granular layer of the thickened atopic dermatitis epidermis no longer localized above nectin-1 and was redistributed to the spinous layer (Fig. 6A). Nectin-1 was still observed on the expanded suprabasal layers, however, the stainings were more diffused (Fig. 6A), which unexpectedly correlates with increased nectin-1 transcript levels compared to controls (14). Costainings with claudin-1 further demonstrated nectin-1 throughout the granular layer with a punctate and reduced pattern of claudin-1 (Fig. 6B) indicative of the strongly redistributed TJ marker in atopic dermatitis epidermis (14). As lesional skin is characterized by epidermal thickening resulting from the altered epidermal growth and keratinocyte terminal differentiation (32), we analyzed potentially redistributed AJs under impaired differentiation conditions and stained E-cadherin. This major AJ component was evenly distributed in the thickened atopic dermatitis epidermis and colocalized with nectin-1 throughout all layers, which was comparable to control skin suggesting that formation of AJs still takes place in the thickened epidermis (Fig. 6C). Our results indicate that impaired differentiation and defective TJs do not influence the presence of nectin-1 throughout the epidermal layers but most likely provide better accessibility of nectin-1 to HSV-1.

**FIG 7:**
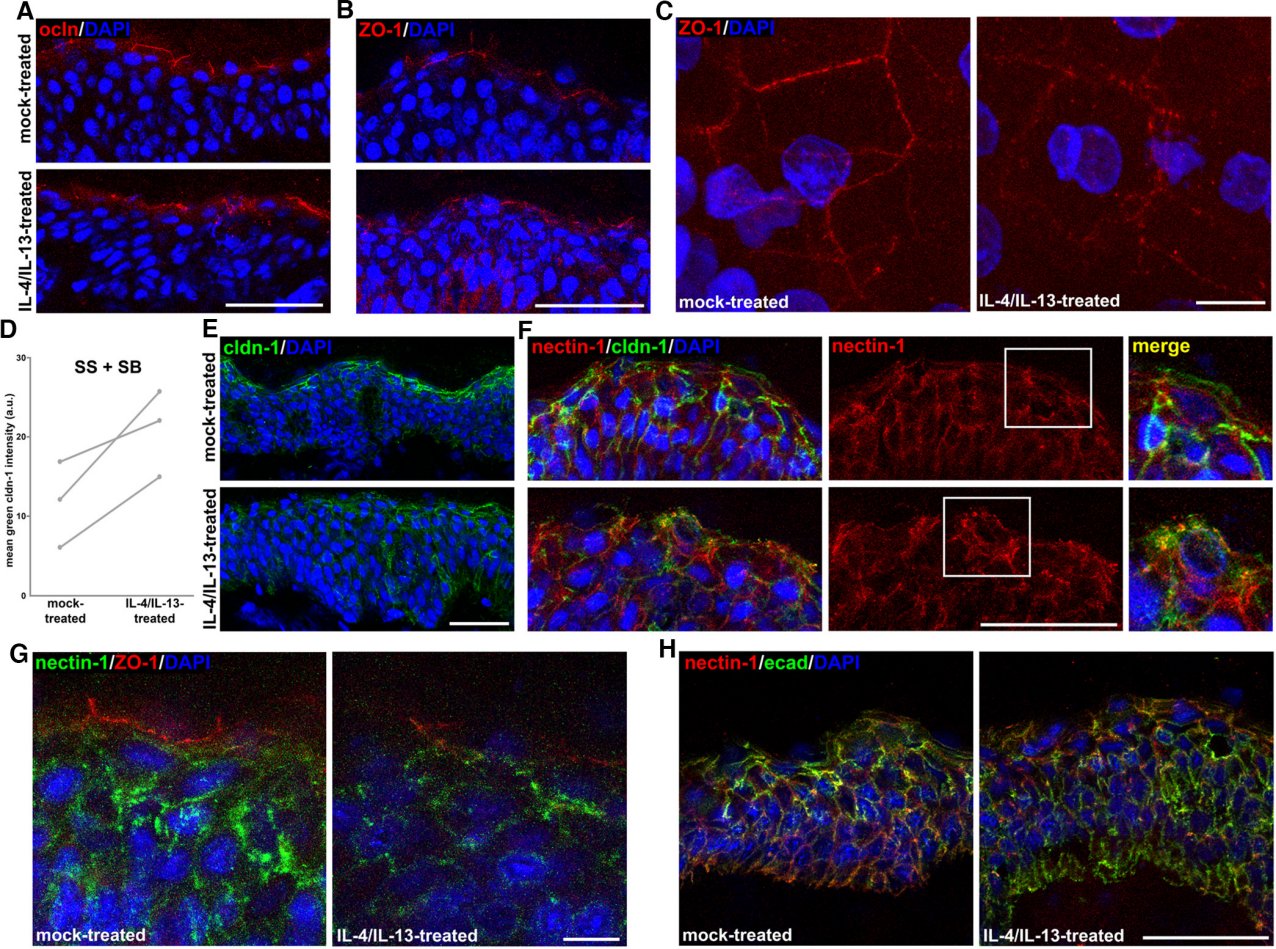
Localization of nectin-1 in IL-4/IL-13-treated human skin. (A) Cross sections depict distinct occludin (ocln; red) in the granular layer of mock-treated skin compared to punctate occludin staining after IL-4/IL-13 treatment (*n* ≥ 3). (B) ZO-1 (red) was present in the granular layer and redistributed to the spinous layer in IL-4/IL-13-treated skin (*n* ≥ 3). (C) Whole mount prepared from human skin after exfoliative toxin-A treatment visualizes the distinct ZO-1 staining (red) in the granular layer of mock-treated skin compared to the punctate ZO-1 staining after IL-4/IL-13 treatment. (D) Quantification of fluorescence shows increased claudin-1 in the stratum spinosum (SS) and the stratum basale (SB) after IL-4/IL-13 treatment (*n* = 3). (E) Cross sections show distinct claudin-1 (cldn-1; green) in mock-treated skin compared to punctate claudin-1 staining after IL-4/IL-13 treatment (*n* ≥ 3). (F) Cross sections demonstrate the redistribution of claudin-1 (cldn-1; green) with regard to nectin-1 (red) in the most apical nucleated cells after IL-4/IL-13 treatment (magnification) and the presence of nectin-1 (red) throughout the epidermal layers of IL-4/IL-13- and mock-treated skin (*n* = 3). (G) Cross sections of the granular layer show nectin-1 (green) underneath ZO-1 (red) in mock- and IL-4/IL-13-treated skin (*n* = 2). (H) Cross sections visualize colocalization of E-cadherin (ecad; green) and nectin-1 (red), which was comparable in mock- and IL-4/IL-13-treated skin. DAPI (blue) serves as a nuclear counterstain. Scale bars, 50 μm (A, B, E), 10 μm (D, C).

To further dissect the parameters that contribute to the viral access of nectin-1 under pathological conditions, we investigated the distribution of TJ and AJ components with regard to the presence of nectin-1 in healthy human skin samples stimulated with IL-4/IL-13. Recent studies demonstrated some penetration of HSV-1 via the skin surface after cytokine induction, although we observed no obvious impairment of the cornified layer (14). Here, we explored potential alterations of TJ components. Occludin, visible in the apical granular layer, showed a punctate staining pattern in IL-4/IL-13-treated skin compared to the distinct pattern in mock-treated skin (Fig. 7A). Although still present in the granular layer, ZO-1 was redistributed to the spinous layer of cytokine-stimulated epidermis (Fig. 7B) and whole-mount preparations visualized the change from a distinct to a punctate staining pattern of ZO-1 in the most apical granular layer (Fig. 7C). The distinct distribution of claudin-1 in mock-treated skin also changed to a punctate staining (Fig. 7E) and showed enhanced redistribution to the spinous and basal layers (Fig. 7D and E). These altered staining patterns support the idea that the IL-4/IL-13 treatment can induce impaired TJs. Costainings indicated relocalization of claudin-1 regarding nectin-1 in the most apical granular layer upon IL-4/IL-13 treatment (Fig. 7F), while we detected nectin-1 underneath ZO-1 both in mock- and cytokine-treated skin (Fig. 7G). As in atopic dermatitis epidermis, we found no obvious redistribution of E-cadherin throughout the IL-4/IL-13-treated skin (Fig. 7H). In summary, the cytokine-induced alterations of TJs and the presence of nectin-1 in the granular layer further supports that HSV-1 can gain access to its receptor just underneath impaired TJs.

## DISCUSSION

As nectin-1 represents the primary receptor for HSV-1 entry, the intriguing question is how this component of AJs can be reached by HSV-1 upon exposure of human skin. Analyses of human skin revealed nectin-1 on 40% to 85% of epidermal cells, including undifferentiated and differentiated keratinocytes (13). While the variable nectin-1 expression correlated neither with age nor with skin area, it is still open whether the distribution throughout the epidermal layers can vary (13). Here, we demonstrated the distribution of nectin-1 in human epidermis and how the formation and impairment of TJ formation in human epidermis correlated with successful HSV-1 invasion which revealed the conditions under which nectin-1 was accessible to the virus. We found that TJ formation in stratified cultures of N/TERT-1 cells interfered with HSV-1 entry and infection of human epidermal equivalents confirmed that the extent of TJ formation during differentiation correlated with the number of infected cells in the suprabasal layers. While mature TJs limited viral invasion, early cornification during the differentiation process provided a further barrier, as uptake of beads as well as viral penetration was restricted. To follow up with studies in an epidermal model closer to *in vivo* skin homeostasis, we adopted epidermal equivalents based on primary human keratinocytes which showed some heterogeneity in their morphology more closely reflecting the situation in skin. During differentiation, cell areas with TJ formation prevented viral penetration strengthening the role of TJs as effective barriers. Further TJ formation and early cornification processes strongly interfered with viral invasion; however, the contribution of the stratum corneum barrier remained vague. Barrier formation of the cornified layer and TJs have an interdependent relationship as cornification results from denucleation of keratinocytes of the granular layer and replacement of the plasma membranes by cornified lipid envelopes still harboring junction components (33). This makes it difficult to assess the contribution of each barrier in restricting HSV-1 invasion. *Ex vivo* infection of murine skin revealed that removal of the stratum corneum was insufficient to allow HSV-1 invasion, emphasizing the role of functional TJs (21).

To further explore the role of functional TJs and the cornified layer, we asked how impairment of epidermal barriers contribute to facilitated viral penetration. We confirmed that induction of Th2 responses by treatment of N/TERT-1 3D cultures results in impaired epidermal barriers (17, 25–27), as latex beads penetrated in the granular layer. However, viral penetration was still restricted, suggesting that additional demands of impaired barriers are needed for successful invasion. The challenge of HSV-1 is to gain access to its receptor nectin-1, implying that the virus must not only penetrate the cornified layer but must also overcome the TJs to reach nectin-1. We then investigated inflammation-induced effects on the early state of TJ formation in 3D cultures based on primary keratinocytes. Our results support that the cytokine responses after short induction by IL-4/IL-13 can already interfere with TJ development which, in turn, correlates with enhanced infection efficiency, suggesting that the Th2 cytokines can target the TJs rather early. Furthermore, enhanced infection was found in epidermal equivalents based on nonlesional atopic dermatitis keratinocytes, which demonstrated an intrinsic predisposition to defective barrier formation and strengthened the role of TJ formation. This finding further supports the idea that defective epidermal barriers of atopic dermatitis skin facilitate HSV-1 infection (14).

Next to barrier formation, the nectin-1 distribution in the epidermal layers plays a major role in understanding the viral access of the receptor. Nectin-1 was strongly present in the basal layer, as expected, and during differentiation, enriched lateral nectin-1 gradually decreased toward the most apical nucleated cells and was visible just underneath TJs. As HSV-1 can penetrate lesional skin of atopic dermatitis patients (14), we explored how these pathological skin conditions influence the presence and/or distribution of the receptor. Nectin-1 was present throughout all epidermal layers of atopic dermatitis skin; however, its distribution toward the TJ markers ZO-1 and claudin-1 strongly changed suggesting that impaired TJs make nectin-1 more accessible to the virus. Impaired TJs next to the well-known dysfunctions of the stratum corneum are implicated in atopic dermatitis (28, 34–36). Furthermore, defective TJs have been shown to modulate the barrier formation of the stratum corneum (37). Thus, we cannot dissect the impact of the individual barriers provided by either the stratum corneum or the TJs but suggest that the abnormal cornified layer of atopic dermatitis skin facilitates viral penetration and that only the impaired TJs allow viral access to its receptor nectin-1.

We also observed viral invasion via the skin surface after IL-4/IL-13 stimulation of human skin from healthy individuals, which was less efficient than in atopic dermatitis skin (14). Comparable to atopic dermatitis skin, claudin-1 was redistributed upon cytokine treatment and occludin and ZO-1 patterns were also changed. Although nectin-1 remained localized underneath ZO-1 as in mock-treated skin, the redistributed TJ components most likely indicate impaired TJ barriers which facilitate receptor accessibility. In contrast to atopic dermatitis skin, we observed no obvious defects of the cornified layer upon cytokine treatment of human skin (14). As reduced claudin-1 might alter the stratum corneum barrier (38, 39), we speculate that minor effects on the barrier function could contribute to limited viral invasion, while dysfunctional TJs led to nectin-1 accessibility.

Next to the physical barriers, Langerhans cells, as major players of the immune barrier, comprise an important element of the skin barrier. As epidermal Langerhans cells can elongate their dendrites to penetrate TJs (40), penetration of these mononuclear phagocytes might offer an alternative pathway to overcome the TJ barrier in epithelia with minor or no cornification. While infection of oral human mucosa or lesional atopic dermatitis skin revealed no preferred infection of Langerhans cells (14, 21), initial infection of various skin mononuclear phagocytes was described in human foreskin epidermis characterized by minor cornification (41). Thus, the role of skin barriers for HSV-1 invasion is, most likely, tightly connected to the type of epithelia and the distribution of nectin-1.

Taken together, we conclude that access of HSV-1 to its receptor nectin-1 in human skin depends on dysfunctional TJs under pathological conditions such as in atopic dermatitis and in cytokine-treated skin which comprise multiple impaired physical barriers.

## MATERIALS AND METHODS

### Cells and human epidermal equivalents.

The human keratinocyte cell line N/TERT-1 (18, 19) was maintained in K-SFM medium (Gibco) containing 0.4 mM CaCl_2_, 25 μg/mL bovine pituitary extract (BPE), 0.2 ng/μL epidermal growth factor (EGF), 100 IU/mL penicillin, and 100 μg/mL streptomycin and was only grown up to 30% confluence to avoid spontaneous differentiation.

Primary keratinocytes isolated from human juvenile foreskin or adult breast skin were cultured on dishes coated with rat-tail collagen I (30 μg/mL) (Corning), maintained in CnT-PRIME epithelial proliferation medium (CELLnTEC), and grown until 70% confluence. Isolates of atopic dermatitis nonlesional keratinocytes (*n* = 2 individuals) were obtained from Ellen van den Bogaard (Radboud University Medical Center, Nijmegen).

For stratified cultures of N/TERT-1 cells, 1.5×10^5^ cells were seeded in K-SFM medium on coverslips coated with rat-tail collagen I (40 μg/mL) (Corning). Stratification was induced 24 h postseeding by switching to differentiation medium by supplementing 1.8 mM Ca^2+^ to DMEM/Ham’s F12 (1:3) (Life Technologies) containing 10% fetal calf serum (FCS; calcium free), penicillin (100 IU/mL), streptomycin (100 μg/mL), epidermal growth factor (EGF) (10 ng/mL), adenine (1.8×10^−4^ M), hydrocortisone (0.5 μg/mL), cholera toxin (10^−10^ M), insulin (5 μg/mL) and ascorbic acid (0.05 mg/mL). Cultures were refreshed with medium every other day and infected at day 1 or 8 post-calcium induction.

Human epidermal equivalents were generated with N/TERT-1 cells or with primary human keratinocytes isolated from skin of healthy individuals or from nonlesional skin of atopic dermatitis patients as described (19, 42). Briefly, 1.5×10^5^ cells were seeded on transwell filters (pore size 0.4 μm) (Life Technologies) coated with rat-tail collagen I (100 μg/mL) (Corning) and grown to confluence in K-SFM medium (Gibco) for N/TERT-1 cells or in CnT-PRIME medium (CELLnTEC) for primary human keratinocytes. After 48 h, culture medium was replaced by 3D differentiation medium (80% CnT-3D barrier) (CELLnTEC)/20% DMEM (Sigma), and submerged culture inserts were grown for ca. 16 h and then lifted to the air-liquid interface for 8 days. Cultures were refreshed with differentiation medium every other day and infected at various times at day 0 or after airlift.

### Preparation of human skin.

For IL treatment, full-thickness skin samples which were taken from patients undergoing breast (*n* = 4 individuals) or plastic surgery (*n* = 3 individuals) were cut in pieces (ca. 4 × 4 mm) after removal of subcutaneous fat (43). After IL-treatment, epidermal whole mounts were prepared by mechanical removal of as much dermis as possible using surgical scissors. The remaining skin was floated onto a 200-μL droplet of exfoliative toxin A (2 mg/mL) (MyBioSource, MBS1223672) diluted in phosphate-buffered saline (PBS) containing 1 mM CaCl_2_ and incubated at 37°C for 35 min in a humidified chamber. After incubation, the cornified and connected granular layers were separated from the underlying epidermal and remaining dermal layers using forceps.

### Ethics statement.

Human skin specimens were obtained after informed consent from all patients. The study was approved by the Ethics Commission of the Medical Faculty, University of Cologne (approval no. 17-481).

### Interleukin treatment.

To induce an atopic dermatitis-like phenotype, human skin samples and human epidermal equivalents were treated with IL-4 and IL-13. Immediately after surgery, skin samples were treated with IL-4 (25 ng/mL) and IL-13 (25 ng/mL) diluted in DMEM/high-glucose/GlutaMAX (Life Technologies) with 10% fetal calf serum (FCS), penicillin (100 IU/mL), streptomycin (100 μg/mL), and 0.05% bovine serum albumin (BSA) for 3 days. Epidermal equivalents derived from N/TERT-1 or primary human keratinocytes were treated with IL-4 (30 and 50 ng/mL, respectively) and IL-13 (30 and 50 ng/mL, respectively) dissolved in differentiation medium with 0.05% BSA (19, 25). IL-4/13 was added to the human epidermal equivalents at day 1 after airlifting. After 7 days of incubation, mock- and IL-4/13-treated N/TERT-1 epidermal equivalents were infected with HSV-1 at ca. 20 PFU/cell for 3 h. After 1 or 3 days of incubation, mock- and IL-4/13-treated epidermal equivalents from primary keratinocytes were infected with HSV-1 at ca. 50 PFU/cell for 2 h before removal of viral suspension, then further incubated for 4 h with a dry top. The medium containing IL-4/13 was refreshed every second day prior to infection.

### Virus.

Infection studies were performed with HSV-1 wild-type (Glasgow) strain 17+ from purified virus preparations obtained from the supernatant of infected BHK cells, as described (43). The calculation of the virus dose was based on the estimated cell number of the apical surface in the human epidermal equivalents at confluence (ca. 2.0×10^5^). HSV-1 was administered to human epidermal equivalents at 37°C defining time point 0.

### Penetration assay.

Sulfate-modified polystyrene, fluorescently labeled latex beads (500 nm) (Sigma) served as a marker for penetration of particles in tissue. Human epidermal equivalents were incubated with beads (2 ×10^9^ beads/sample) for 3 h or 24 h at 37°C. Samples were thoroughly washed three times and immediately embedded for preparation of cryosections.

### Histochemistry, immunocytochemistry, and antibodies.

For hematoxylin and eosin (H&E) stains, human epidermal equivalents were fixed with 3.4% formaldehyde for 10 min at room temperature (RT) (day 0), 30 min at room temperature (day 2), or overnight at 4°C (day 4 to 8), and fixed samples were prepared as paraffin sections (8 μm). The morphology of the developing epidermal equivalents was assessed by H&E stains.

Stratified cultures and cells seeded on coverslips were fixed with 2% formaldehyde for 10 min at room temperature and permeabilized with 0.5% NP-40 (Sigma) in PBS for 10 min for staining of F-actin or left unpermeabilized for staining of nectin-1, occludin, and ZO-1.

For cryosections, epidermal equivalents and skin samples were embedded in OCT compound (Sakura), frozen at −80°C, and cut into 8-μm-thick cross sections (43). Cryosections were fixed with 2% formaldehyde for 10 min at RT, and epidermal whole-mount preparations of epidermal equivalents were fixed with 3.4% formaldehyde for 10 min at room temperature (day 0), 30 min at room temperature (day 2), or overnight at 4°C (day 4 to 8) (43). For stainings of nectin-1, claudin-1, occludin, and ZO-1, cryosections were fixed with ice-cold ethanol for 30 min and then with acetone (–20°C) for 3 min. Sections of skin and epidermal equivalents were incubated with primary antibodies overnight at 4°C followed by incubation with the species-specific Alexa Fluor-conjugated secondary antibodies and 4′,6-diamidino-2-phenylindole (DAPI) for 45 min at RT. Whole mounts of epidermal equivalents were incubated with primary antibodies overnight at RT and with the secondary antibodies and DAPI overnight at 4°C. The following primary antibodies were used: mouse anti-ICP0 (MAb 11060; 1:60) (44), rabbit anti-loricrin (1:1000; BioLegend), mouse anti-claudin-1 (1:500; A9; Santa Cruz), mouse anti-filaggrin (1:500; AKH1; Santa Cruz), mouse anti-occludin (1:400; OC-3F10; Thermo Fisher Scientific), mouse anti-E-cadherin (1:400; BD-610181; BD Biosciences), rabbit anti-ZO-1 (1:400; Thermo Fisher Scientific), and mouse anti-nectin-1 antibody (1:250 for whole mounts, 1:500 for cryosections; CK41) (45). F-actin was labeled with phalloidin-Atto 565 (1:2000) (Sigma) for 45 min at room temperature.

Microscopy was performed using an epifluorescence microscope (Zeiss Axiophot) equipped with a Nikon Digital Sight camera system (DS-2MV)/NIS Elements software (for H&E stains) and a Leica DM IRBE microscope linked to a Leica TCS-SP/5 confocal unit. Images were assembled using Photoshop (Elements 2018; Adobe) and Illustrator (version CS5; Adobe). Confocal projections and merged images are shown. Images were analyzed using Fiji (version 2.0.0-rc-65/1.51s) (46) by measuring the mean fluorescence intensity of three different areas per sample. XZ/YZ orthogonal and 3D projections of whole mounts were generated using Fiji (version 2.0.0-rc-65/1.51s) and Imaris X64 9.5.1 (Oxford Instruments Group), respectively.

### Statistics.

For the statistical analyses Student's *t* tests were performed to calculate *P* values using the unpaired two-tailed method. Differences were considered statistically significant with *P* values ≤ 0.05 (*).

### Flow cytometric analysis.

Epidermal equivalents (day 8) based on N/TERT-1 cells were incubated with TrypLE Select (Life Technologies) and processed as described (7). Dissociation of the epidermal equivalents resulted in the dissociation of basal and suprabasal cells; apical granular cells were not dissociated and still connected to the cornified layer as shown by H&E stains (data not shown). Cell suspensions were incubated in PBS-5% FCS on ice for 30 min with mouse anti-nectin-1 (CK41; 1:100) (45) and visualized with anti-mouse IgG-Cy5 (1:100) (Jackson Immunoresearch Laboratories Inc.). Mouse IgG1 (Life Technologies, 1:20) was used as isotype control.
